# Supplementary material for: Identification of photoperiod-induced specific miRNAs in the adrenal glands of Sunite sheep (Ovis aries)
Source: Front Vet Sci. 2022 Jul 22;9:888207. doi: 10.3389/fvets.2022.888207 (PMC9354845; doi:10.3389/fvets.2022.888207)
Supplement: Supplementary file 1 [file Data_Sheet_1.ZIP › Supplementary file/miRDeep2 file.zip!/miRDeep2 file/Know_Structure/SP42A1/oar-mir-369.pdf]

miRBase precursor : oar-mir-369
Total read count : 9404
oar-miR-369-5p read count : 701
oar-miR-369-3p read count : 8702
remaining reads : 1

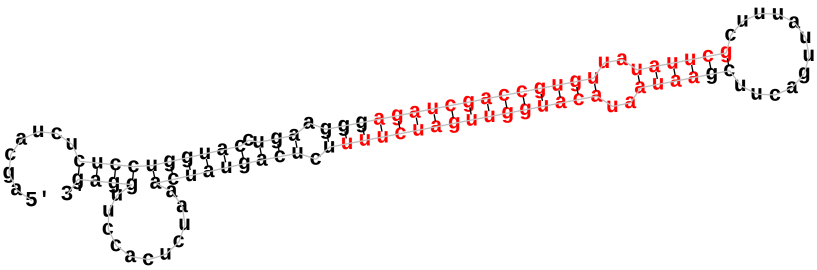

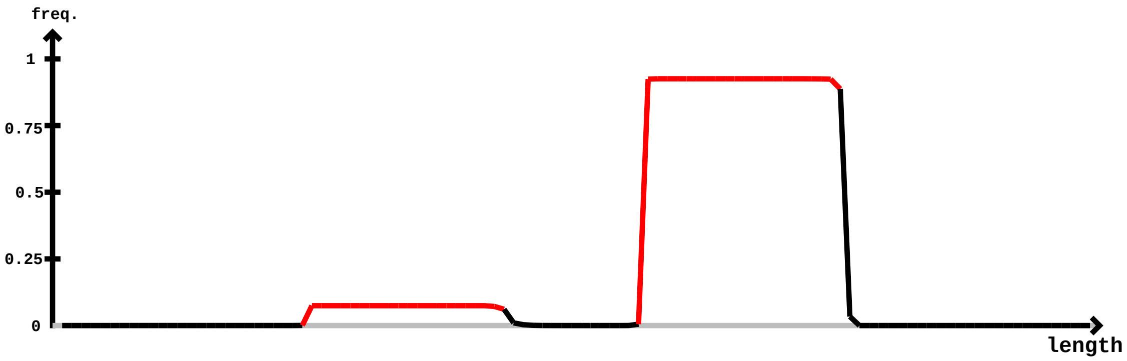


```
                                 oar-miR-369-5p
                                                              oar-miR-369-3p
5'- agcaucucuccugguaccugaagggagaucgaccguguuauauucgcuuuauugacuucgaauaauacaugguugaucuuuucucaguaucaaaucucaccuuggag   -3'    exp
    .......((((((((((.(((.((((((((((((((((..((((((............))))))..)))))))))))))))).)))))))))...........))))    reads    mm    sample
    .....cucuccugguaccugaaggg..................................................................................    1        0     seq
    .......................gagaucgaccguguuauauucg.............................................................    4        0     seq
    .........................agaucgaccguguuauauu...............................................................    28       0     seq
    .........................agaucgaccguguuauauuc..............................................................    98       0     seq
    .........................agaucgaccguguuauauucg.............................................................    475      0     seq
    .........................agaucgaccguguuauauucgc............................................................    65       0     seq
    .........................agaucgaccguguuauauucgcu...........................................................    23       0     seq
    .........................agaucgaccguguuauauucgcuu..........................................................    6        0     seq
    .........................agaucgaccguguuauauucgcuuu.........................................................    1        0     seq
    .........................agaucgaccguguuauauucgcuuua........................................................    1        0     seq
    ..........................................................cgaauaauacaugguugaucuuu..........................    2        0     seq
    ...........................................................gaauaauacaugguugaucuu...........................    3        0     seq
    ...........................................................gaauaauacaugguugaucuuu..........................    45       0     seq
    ............................................................aauaauacaugguugau..............................    1        0     seq
    ............................................................aauaauacaugguugauc.............................    3        0     seq
    ............................................................aauaauacaugguugaucu............................    9        0     seq
    ............................................................aauaauacaugguugaucuu...........................    343      0     seq
    ............................................................aauaauaAaugguugaucuuu..........................    3        1     seq
    ............................................................aauaauacaugguugaucuuu..........................    7968     0     seq
    ............................................................aauaauacaugguugaucuuuu.........................    313      0     seq
    .............................................................auaauacaugguugaucuuu..........................    8        0     seq
    .............................................................auaauacaugguugaucuuuu.........................    4        0     seq
```
